# The *Arabidopsis thaliana* Mediator subunit MED8 regulates plant immunity to *Botrytis Cinerea* through interacting with the basic helix-loop-helix (bHLH) transcription factor FAMA

**DOI:** 10.1371/journal.pone.0193458

**Published:** 2018-03-07

**Authors:** Xiaohui Li, Rui Yang, Haimin Chen

**Affiliations:** Key Laboratory of Applied Marine Biotechnology, Ningbo University, Ningbo, Zhejiang, China; National Taiwan University, TAIWAN

## Abstract

The Mediator complex is at the core of transcriptional regulation and plays a central role in plant immunity. The MEDIATOR25 (MED25) subunit of *Arabidopsis thaliana* regulates jasmonate-dependent resistance to *Botrytis cinerea* through interacting with the basic helix-loop-helix (bHLH) transcription factor of jasmonate signaling, MYC2. Another Mediator subunit, MED8, acts independently or together with MED25 in plant immunity. However, unlike MED25, the underlying action mechanisms of MED8 in regulating *B*. *cinerea* resistance are still unknown. Here, we demonstrated that MED8 regulated plant immunity to *B*. *cinerea* through interacting with another bHLH transcription factor, FAMA, which was previously shown to control the final proliferation/differentiation switch during stomatal development. Our research demonstrates that FAMA is also an essential component of *B*. *cinerea* resistance. The *fama* loss-of-function mutants (*fama-1* and *fama-2*) increased susceptibility to *B*. *cinerea* infection and reduced defense-gene expression. On the contrary, transgenic lines constitutively overexpressing *FAMA* showed opposite *B*. *cinerea* responses compared with the *fama* loss-of-function mutants. *FAMA*-overexpressed plants displayed enhanced resistance to *B*. *cinerea* infection and increased expression levels of defensin genes following *B*. *cinerea* treatment. Genetic analysis of MED8 and FAMA suggested that FAMA-regulated pathogen resistance was dependent on MED8. In addition, MED8 and FAMA were both associated with the G-box region in the promoter of *ORA59*. Our findings indicate that the MED8 subunit of the *A*. *thaliana* Mediator regulates plant immunity to *B*. *cinerea* through interacting with the transcription factor FAMA, which was discovered to be a key component in *B*. *cinerea* resistance.

## Introduction

*B*. *cinerea* is a ubiquitous pathogen that causes gray mold disease on more than 200 host plants and results in crop losses of up to 20% globally [1]. As a typical necrotrophic pathogen, *B*. *cinerea* can produce a variety of cell wall-degrading enzymes, phytotoxic metabolites, and cell death elicitors to destroy host cells and induce necrosis [2, 3]. In order to defend *B*. *cinerea* attack, plants have evolved a complex immune system including changes in ion fluxes, synthesis of the defense related hormones, and transcriptional reprogramming [4–7]. The precise transcriptional regulation of a wide range of genes encoding diverse molecules is pertinent in determining plant resistance and susceptibility to *B*. *cinerea* infection [1]. Recent studies have indicated that the Mediator complex plays an important role in the transcriptional process underpinning plant immunity to bacterial and fungal infection.

Mediator is a conserved multisubunit complex which connects the transcription factors located in the promoter regions of protein-coding genes to the RNA polymerase II (Pol II) at the transcription start site in eukaryotes [8]. The *Arabidopsis* Mediator complex contains 21 conserved and 6 plant-specific subunits [9]. A number of mediator subunits play critical roles in a variety of signaling pathways including growth and development, response to biotic and abiotic stress, and cell life activities such as noncoding RNA processing, adjusting the stability of DNA and proteins, and secondary metabolism [10–24]. Among them, MED8, MED16, MED18, MED21, MED25, and CDK8 play significant roles in plant immunity to necrotrophic pathogens [10, 11, 16, 17, 20, 21]. MED16 together with transcription factor WRKY33 were found to be critical to basal resistance against another devastating necrotrophic fungal plant pathogen in agriculture, *Sclerotinia sclerotiorum* [25]. MED18 interacts with the transcription factor YIN YANG1 to suppress the disease susceptibility genes glutaredoxins *GRX480* and *GRXS13*, and thioredoxin *TRX-h5* to mediate plant immunity to *B*. *cinerea* [20]. MED21 interacts with the *A*. *thaliana* RING E3 ligase HUB1, and *MED21* RNAi plants are highly susceptible to *A*. *brassicicola* and *B*. *cinerea* infection [10]. CDK8 was found to regulate cuticle development by interacting with the transcription factor WAX INDUCER1, and the *cdk8* mutant exhibited enhanced resistance to *B*. *cinerea* [21]. MED25 physically associates with the transcription factor MYC2 in the promoter regions of MYC2 target genes and exerts a positive effect on MYC2-regulated gene transcription during JA-dependent plant immunity [16]. As with MED25, MED8 is a regulator of JA-dependent plant immunity. The *med8* mutant exhibited an *F*. *oxysporum* resistance phenotype and had increased susceptibility to *A*. *brassicicola* [11]. In addition, the expression level of *PDF1*.*2* was slightly lower in both untreated and MeJA-treated *med8* plants than in untreated and MeJA-treated wild-type plants. This suggests that MED8 is important to JA-dependent plant immunity. The *med8med25* double mutant exhibited stronger defense than either of the single mutants, suggesting that MED8 and MED25 probably affect JA-dependent plant immunity signaling by independent and additive mechanisms[11].

FAMA, a basic helix-loop-helix (bHLH) transcription factor, was first reported to control the final proliferation/differentiation switch during stomatal development [26–30]. Three bHLH transcription factors including SPEECHLESS (SPCH), MUTE, and FAMA were found to regulate stomatal differentiation that proceeds through a series of steps originating from meristemoid mother cells [31–36]. SPEECHLESS is required for the first asymmetric ‘entry’ division into the stomatal lineage and is involved in promoting the asymmetric ‘amplifying’ divisions of meristemoids [37]. MUTE is essential for the termination of the stem cell-like asymmetric division activity and promotion of differentiation [38]. The FAMA transcription factor regulating the later stages of stomatal development is necessary to prevent further mitotic division of the guard mother cell after the single division that normally gives rise to a guard cell pair, and promotes guard cell fate [30]. In addition to its function in stomatal development, FAMA is an essential component for the differentiation of myrosin cells. Myrosin cell development and the biosynthesis of the myrosinases THIOGLUCOSIDE LUCOHYDROLASE1 (TGG1) and TGG2 were changed in both *fama* mutant and *FAMA-*overexpressed plants. *FAMA* expression in myrosin cells is independent of SPCH and MUTE [39, 40].

In this study, a yeast two-hybrid assay was performed to identify the potential transcription factors interacting with MED8. FAMA was identified as a potential partner of MED8 in plant immunity towards *B*. *cinerea* and was found to positively influence plant resistance to *B*. *cinerea* infection. The evidence showed that both FAMA and MED8 could be recruited to the G-box region in the promoter of the pathogen-related gene *ORA59*. In addition, we revealed that FAMA and MED8 functioned in the same pathway of plant immunity to *B*. *cinerea* based on genetic evidence. Our study not only elucidates the molecular mechanisms underlying MED8-regulated plant immunity to *B*. *cinerea*, but also extends our understanding of the biological functions of FAMA in regulating plant immunity.

## Materials and methods

### Plant materials and growth conditions

*Arabidopsis thaliana* ecotype Col-0 was used as the wild-type (WT). The mutant lines used are listed in the accession numbers section at the end of the methods. Homozygous lines including *med8*, *med25*, *fama-1*, and *fama-2* were identified using the primers provided at http://signal.salk.edu/tdnaprimers.html and were used in the experiments described below. *med8med25*, *med8fama-2*, *ProMED8*: *MED8-GFP/fama-2*, and *ProFAMA*: *FAMA-GFP/med8* were generated by crossing the parental single homozygous lines. The resulting F2 segregating progenies were genotyped to identify homozygous plants.

To obtain seeds, *A*. *thaliana* plants were grown in Murashige and Skoog (MS) medium [41] or sterile soil in plastic trays at 22°C with a 16-h-light/8-h-dark photoperiod (light intensity 120 μM photons m^−2^ s^−1^) as previously described [16]. For *B*. *cinerea* inoculation, *A*. *thaliana* plants were grown in sterile soil at 22°C with a 12-h-light/12-h-dark photoperiod as previously described [42]. *Nicotiana benthamiana* was grown in sterile soil under a 16-h-light (28°C)/8-h-dark (22°C) photoperiod.

### DNA constructs and plant transformation

The promoters of *MED8* and *FAMA* were amplified with the listed primers (S1 Table). Enzyme-digested PCR products were cloned into the same site of the pCAMBIA1300 vector to generate *ProMED8*: *pCAMBIA1300* and *ProFAMA*: *pCAMBIA1300*, respectively. Full-length coding sequences of *MED8* and *FAMA* were amplified with the listed primers (S1 Table) and the enzyme-digested PCR products were ligated with *ProMED8*: *pCAMBIA1300* and *ProFAMA*: *pCAMBIA1300* to generate *ProMED8*: *MED8-GFP* and *ProFAMA*: *FAMA-GFP*. All primers used for DNA construct generation are listed in S1 Table.

The constructs of *ProMED8*: *MED8-GFP* and *ProFAMA*: *FAMA-GFP* were transformed into *Agrobacterium* strain GV3101 (pMP90), which was used for the transformation of the *A*. *thaliana* plants via the floral dip method [43]. Transformants were selected based on their resistance to hygromycin (25 μg/mL). Homozygous T3 or T4 transgenic seedlings were used for phenotyping and molecular characterization.

### Yeast two-hybrid (Y2H) assays

Y2H assays were performed to assess the interaction of the transcription factors *MYC2* and *FAMA* with *MED8* and *MED25*. Full-length coding sequences of *MED8* and *MED25* were amplified using the listed primers for Y2H assays (S1 Table). Enzyme-digested PCR products were cloned into the same site of pGBKT7. Full-length *MYC2* and *FAMA* were also amplified with the listed primers (S1 Table) and cloned into pGADT7. Matchmaker GAL4 two-hybrid systems (Clontech, Mountain View, California, USA) were used following the manufacturer’s instructions. Constructs for testing the interactions were co-transformed into the yeast strain *Saccharomyces cerevisiae* AH109. Transgene presence was confirmed by growth on an SD/-Leu/-Trp plate. To assess protein interactions, the transformed yeasts were suspended in liquid SD/-Leu/-Trp to OD = 1.0. Five microliters of suspended yeast were placed into the wells of 96-well plates containing SD/-Ade/-His/-Leu/-Trp medium. The interactions were observed after 3 d of incubation at 30°C. The experiments were performed in triplicate.

### Co-immunoprecipitation (Co-IP) assays

The full-length coding sequence of *MED8* was amplified using Gateway-compatible primers (S1 Table). The PCR product was cloned using pENTR Directional TOPO cloning kits (Invitrogen, Carlsbad, USA) and then recombined with the binary vector PGWB5 (35S promoter, C-GFP) to generate the *35Spro*:*MED8-GFP* construct. The full-length coding sequence of *FAMA* was also cloned into the pGWB14 vector (35S promoter, C-3HA) to generate the *35Spro*: *FAMA-HA* construct. The constructs of *35Spro*:*MED8-GFP* and *35Spro*: *FAMA-HA* were transformed into Agrobacterium strain GV3101 (pMP90). Then agrobacterial strains carrying constructs of *35Spro*:*MED8-GFP* and *35Spro*:*FAMA-HA* were co-infiltrated into *Nicotiana benthamiana* leaves. Agrobacterial strains carrying constructs of *GFP-myc* were used as a control. The infiltrated parts of *N*. *benthamiana* leaves were harvested and then ground in liquid nitrogen and re-suspended in extraction buffer (50 mM Tris-HCl, pH 7.5, 150 mM NaCl, 0.1% Triton X-100, 0.2% Nonidet P-40, 0.6 mM PMSF, and 20 μMMG132 with Roche protease inhibitor cocktail). After protein extraction, 20 μL protein G plus agarose (Santa Cruz Biotechnology, Dallas, TX, USA) was added to the 2-mg extracts to reduce nonspecific immunoglobulin binding. After 1 h of incubation, the supernatant was transferred to a new tube. GFP antibody-bound agarose beads (MBL) were then added to each reaction for 1 h at 4°C. The precipitated samples were washed at least three times with the lysis buffer and then eluted by adding 1×SDS protein loading buffer with boiling for 5 min. Total and immunoprecipitated proteins were analyzed by immunoblotting using anti-HA and anti-GFP antibodies.

### Chromatin immunoprecipitation (ChIP)-PCR assays

*ProFAMA*: *FAMA-GFP*, *ProMED8*: *MED8-GFP*, *ProFAMA*: *FAMA-GFP/med8*, *ProMED8*: *MED8-GFP/fama-2* or wild type seedlings were grown in MS medium. For *B*. *cinerea* treatment, 5-d-old seedlings of the above materials were sprayed with 0.5×10^5^ to 1.0×10^5^ spores/mL of *B*. *cinerea* over a period of 36 h, after which 1.5 g of inoculated entire plants were collected. Additionally, 1.5 g of uninoculated seedlings were collected as a control. The collected seedlings were cross-linked in 1% formaldehyde, and their chromatin isolated [44]. A GFP antibody (Abcam, Cambridge, UK) was used to immunoprecipitate the protein-DNA complex, and the precipitated DNA was purified using a PCR purification kit (Qiagen, Hilden, Germany) in preparation for real-time quantitative (RT-qPCR) analysis. The ChIP experiments were performed three times. Chromatin precipitated without an antibody constituted the negative control, while the isolated chromatin prior to precipitation was used as an input control. Primers used for ChIP-PCR are listed in S1 Table.

### RNA extraction and gene expression analyses

To quantify the *FAMA* transcript levels in the *fama* mutants, total RNA was extracted from 2-week-old seedlings grown in MS medium. To evaluate the expression level of *FAMA* in *FAMA* overexpression plants (*OE3* and *OE7*), total RNA was extracted from 6-d-old entire plants grown in MS medium. For the quantitative analysis of the expression levels of pathogen-responsive genes in *fama* mutants plants, total RNA was extracted from 3-week-old seedlings sprayed with *B*. *cinerea* as described below. For the quantitative analysis of the expression levels of pathogen-responsive genes in *med8*, *med25*, *FAMA* overexpression (*OE3* and *OE7*) and *OE-7/ med8* plants, total RNA was extracted from 5-week-old seedlings sprayed with *B*. *cinerea* as indicated (using TRIzol [Invitrogen] reagent). For small-scale RNA isolation, total RNA was extracted using the RNAqueous kit (Ambion, Foster City, California, USA). cDNA was prepared from 2 μg of total RNA with Superscript III reverse transcriptase (Invitrogen) and quantified with a cycler apparatus (Roche 480) using the SYBR Green kit (Takara, Japan) according to the manufacturer’s instructions. Expression levels of target genes were normalized to ACTIN7. The RT-qPCR experiments were performed three times. The statistical significance was evaluated by Student’s *t*-test. Primers used for RT-qPCR are listed in S1 Table.

### Disease assays

*Botrytis cinerea* strain *B05-10* was cultured on 2×V8 (36% V8 juice, 0.2% CaCO_3_, 2% Bacto-agar) and incubated at 22°C [45]. In the detached leaf disease assays of the *fama* mutants, a single 2.5 μL drop of a suspension of 0.5×10^5^ spores/mL in 1% Sabouraud Maltose Broth buffer was placed onto the detached leaves of *fama* mutants and Col-0 plants that had been grown for 3 weeks in growth chambers as described earlier. The detached leaves were placed in transparent trays, which were sealed to maintain high humidity. Disease lesion diameter was measured after 2 d. For the whole plant disease assays of the *fama* mutants, the two mutants plants along with the Col-0 plants were inoculated by spraying with a suspension of 0.5×10^5^ spores/mL in 1% Sabouraud Maltose Broth buffer. After inoculation, the plants were sealed with a transparent cover to maintain high humidity. Fungal hyphal staining and fungal growth were measured after 2 d. For the detached leaf disease assays for *med8*, *med25*, *OE-3*, *OE-7*, and *OE-7/ med8* plants, a single 2.5 μL drop of a suspension of 2.5×10^5^–3×10^5^ spores/mL in 1% Sabouraud Maltose Broth buffer was placed onto the detached leaves of plants grown for five weeks. Once again, high humidity was maintained by sealing the transparent trays. Disease lesion diameter was measured after 3 d. For the whole plant disease assays, the above plants along with the Col-0 plants were inoculated by spraying with a suspension of 2.5×10^5^–3×10^5^ spores/mL in 1% Sabouraud Maltose Broth buffer. After inoculation, the plants were sealed with a transparent cover to maintain high humidity. Fungal hyphae staining and fungal growth was assessed after 3 d. All disease assays were repeated at least in triplicate.

### Fungal hyphal staining with trypan blue

In order to visualize the fungal hyphae after *B*. *cinerea* inoculation, whole leaf mounts were stained with lactophenol-trypan blue (10 mL of lactic acid, 10 mL of glycerol, 10 g of phenol, 10 mg of trypan blue, dissolved in 10 mL of distilled water) as previously described [46]. Whole leaves were boiled for approximately 2 min in the staining solution and then decolorized in chloral hydrate (2.5 g of chloral hydrate dissolved in 1 mL of distilled water) for at least 30 min. They were then mounted in chloral hydrate and viewed under a compound microscope equipped with interference or phase-contrast optics.

### Transient expression assay in *N*. *benthamiana* leaves

The transient expression assays were performed in *N*. *benthamiana* leaves as previously described [47]. For split-luciferase complementation (Split-LUC) assay, *MED8* was cloned into vector pCAMBIA1300-nLUC, and *FAMA* was cloned into vector pCAMBIA1300-cLUC. Primers are summarized in S1 Table. For transcriptional activation assays, the *ORA59* promoter was amplified using Gateway-compatible primers. The PCR products were cloned by pENTR Directional TOPO cloning kits (Invitrogen) and recombined with the binary vector pGWB35 to generate the reporter construct *ORA59*_*pro*_: *LUC*. The *FAMA* effector construct was *35S*_*pro*_: *FAMA-GFP* (*35S*_*pro*_: *FAMA*). We used a low-light cooled CCD imaging apparatus (NightOWL II LB983 with indigo software) to capture the LUC image and to assess luminescence intensity. The leaves were sprayed with 0.5 mM luciferin and placed in darkness for 3 min prior to luminescence detection.

### Microscopy

For two-channel fluorescence imaging using GFP and propidium iodide (PI) fluorescence filter sets, 6-d-old fresh seedlings of *OE-3* and *OE-7* were immersed in 2mg/mL PI solution for 5 min and then rinsed briefly with water before visualization with the Nikon microscope.

### Accession numbers

Sequence data from this article can be found in the *Arabidopsis* Genome Initiative or GenBank/EMBL databases under the following accession numbers: *MED25* (AT1G25540), *ORA59* (AT1G06160), *MED8* (At2g03070), *PDF1*.*2* (At5g44420), *ERF1* (AT3G23240), *PR1* (AT2G14610), *ACTIN7* (At5g09810), *FAMA* (AT3G24140). The following mutant lines were used: *med8* (At2g03070, SALK_092406), *med25* (AT1G25540, SALK_129555), *fama-1* (AT3G24140, SALK_100073), *fama-2* (AT3G24140, SALK_049126).

## Results

### MED8 affects plant immunity to *B*. *cinerea* via a pathway other than MED25

**Fig 1:**
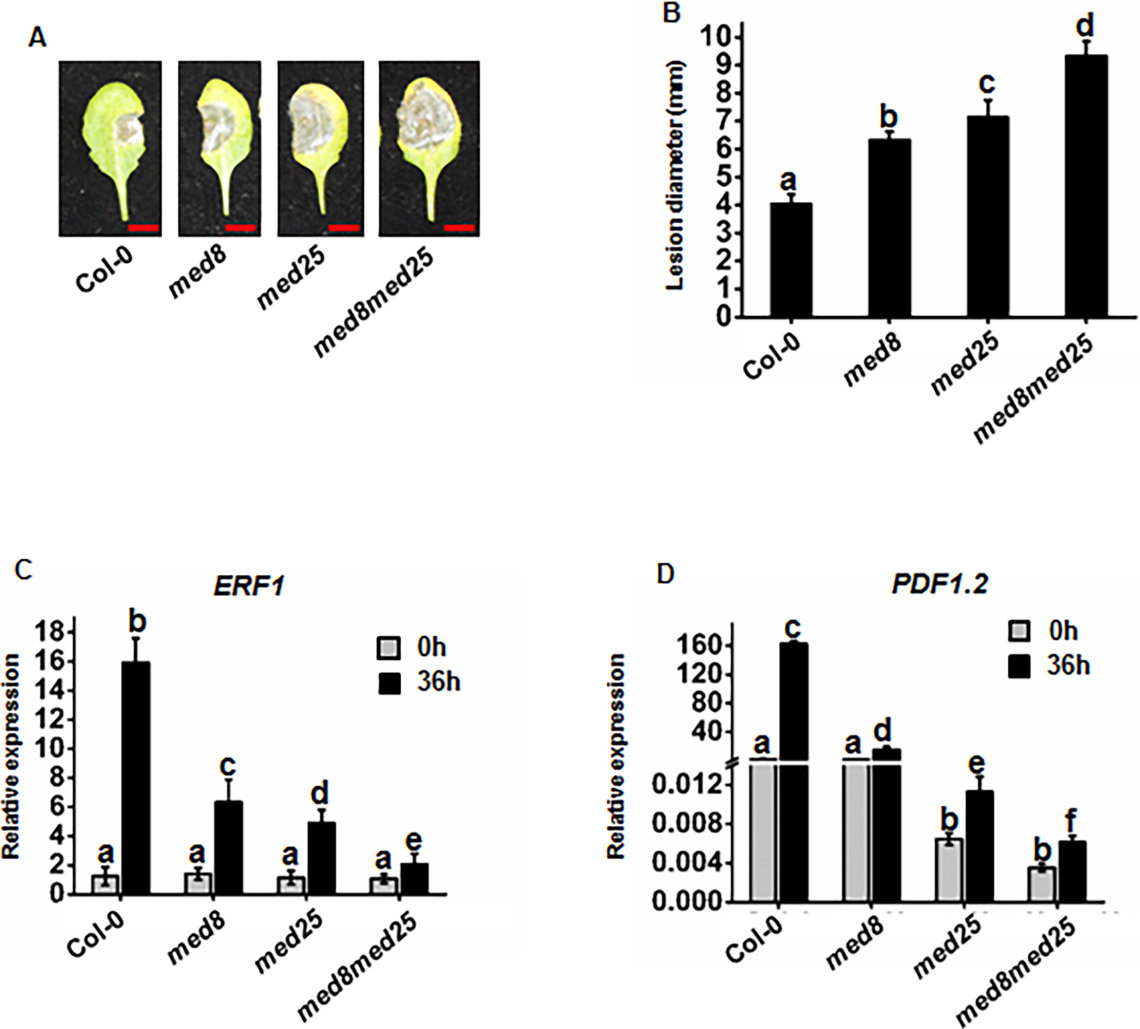
*med8* and *med25* mutants affect plant resistance to *B*. *cinerea* by independent and additive means. (A) (B) Disease symptoms and lesion sizes on the leaves of the *B*. *cinerea*-infected WT, *med8*, *med25*, and *med8med25* at 3 d. The disease assay was performed by drop inoculation of *B*. *cinerea* on the leaves of soil-grown plants. The infected leaves were photographed and bar = 4 mm (A). Average values and SEM from relative values obtained in four biological replicates were plotted on the graph (B). A minimum of 10 leaves for each genotype was used for each biological replicate, and the disease assay was repeated at least four times, with similar results. The mean values followed by different letters represent significant differences (*P*< 0.01, Student’s *t*-test). (C) (D) RT-qPCR analysis of *ERF1* and *PDF1*.*2* RNA levels in the WT, *med8*, *med25*, and *med8med25* leaves of soil-grown plants at 36 hpi after inoculation with *B*. *cinerea*. Expression of *ERF1* and *PDF1*.*2* was normalized against the constitutively expressed *Actin7*. Average values and SEM from relative values obtained in four biological replicates were plotted on the graph. The mean values followed by different letters represent significant differences (*P*< 0.01, Student’s *t*-test).

Previous research demonstrated that *med8* and *med25* mutants influence jasmonic acid (JA)-induced pathogen resistance by independent and additive mechanisms [11]. Chen et al. [16] also reported that MED25 regulates JA-dependent pathogen resistance by interacting with the transcription factor MYC2. However, the details regarding the role of MED8 in resistance to *B*. *cinerea* remain unknown. To elucidate the role of MED8 in *B*. *cinerea* resistance, the disease phenotype of the T-DNA insertion (SALK_092406; [11]; [16]) mutant of *MED8* needed to be verified. We thus used detached leaf disease assays to rapidly evaluate the disease phenotype in *med8* mutant plants in comparison to the WT plants. Typical disease lesions were observed 3 d post-inoculation (dpi). The lesions on the *med8* mutant plant leaves were larger than the WT plants at 3 dpi, and an approximately 38% increase in lesion size was observed (Fig 1A and 1B). Similar enhanced susceptibility was observed in the *med25* mutant plants, and MED8 also possessed an additive effect with MED25 in pathogen susceptibility (Fig 1A and 1B) [11]. Pathogen responsive gene expression was altered in the *med8* mutant. Two marker genes *ERF1* and *PDF1*.*2* [48], considered to be associated with the plant response to pathogen infection [49, 50], were selected for comparison of expressional changes in the *med8* plants with the WT plants following *B*. *cinerea* infection. No significant differences in expression were observed in the two pathogen marker genes in the uninfected *med8* mutant compared with the WT plants, indicating that the pathogen responsive genes were not constitutively expressed in the *med8* mutant. In comparison to the mock-treated plants, the expression levels of *ERF1* and *PDF1*.*2* increased significantly following *B*. *cinerea* infection. Interestingly, the expression levels of *ERF1* and *PDF1*.*2* in the *med8* mutant plants were remarkably reduced in comparison to the WT plants. Additionally, the expression levels of *ERF1* and *PDF1*.*2* in the *med25* mutant plants were significantly reduced compared to that in the *med8* mutant plants (Fig 1C and 1D). This indicates that MED25 plays a more important role in the expression of *ERF1* and *PDF1*.*2* than MED8. Furthermore, the *med8med25* double mutant exhibited lower *ERF1* and *PDF1*.*2* expression levels compared to the *med8* or *med25* single mutant, which suggests that MED8 had an additive effect with MED25 in inducing *ERF1* and *PDF1*.*2* expression. These findings are consistent with previous results whereby the expression levels of pathogen-induced defense genes in *med8med25* double mutant plants were lower than those in *med8* or *med25* single mutants [11]. Ultimately, the results demonstrate that MED8 affects plant immunity to *B*. *cinerea* is a similar manner to MED25. The stronger effect of the *med8med25* double mutant on pathogen resistance than either of the single mutants suggests that the *med8* and *med25* mutations are likely to affect pathogen resistance by independent and additive mechanisms.

### MED8 and FAMA have a direct physical interaction

**Fig 2:**
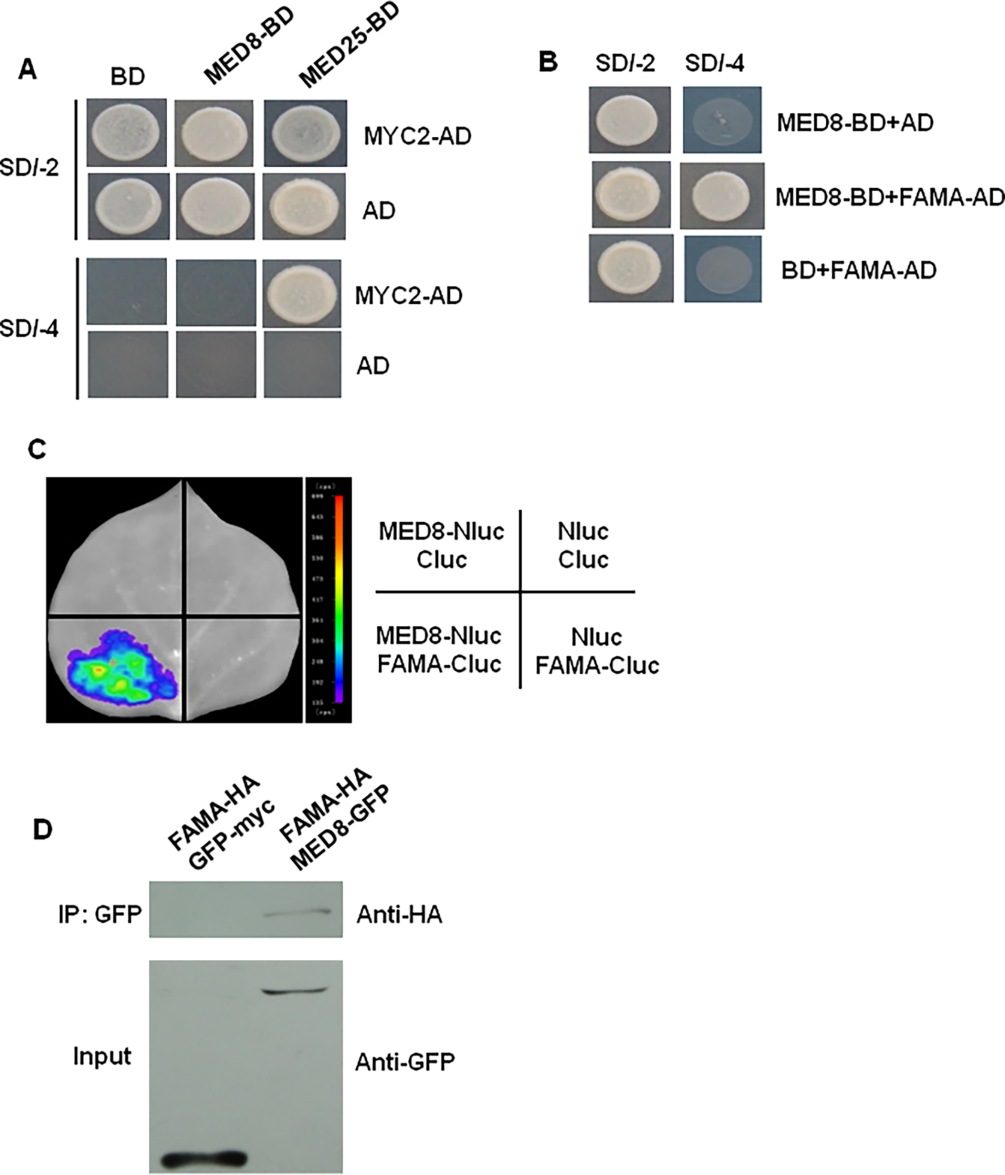
MED8 can interact with FAMA, but not with MYC2. (A) A Y2H assay was used to detect the interactions of MED8 with the MYC2 protein. Yeast cells co-transformed with pGADT7-MYC2 (preys) and pGBKT7-MED8 (baits) were selected and subsequently grown on yeast synthetic dropout lacking Leu and Trp (SD/-2) as a transformation control, or on selective media lacking Ade, His, Leu, and Trp (SD/-4) to test protein interactions. The pGADT7-MYC2 (preys) and pGBKT7-MED25 (baits) interaction constituted a positive control. pGADT7-MYC2 co-transformed with the pGBDT7 vector, and pGBKT7-MED8 or pGBKT7-MED25 co-transformed with the pGADT7 vector were included as controls. (B) A Y2H assay used to detect the interactions of MED8 with the FAMA protein. Yeast cells co-transformed with pGADT7-FAMA (preys) and pGBKT7-MED8 (baits) were selected and subsequently grown on yeast synthetic dropout lacking Leu and Trp (SD/-2) as a transformation control, or on selective media lacking Ade, His, Leu, and Trp (SD/-4) to test protein interactions. pGADT7-FAMA co-transformed with the pGBDT7 vector, and pGBKT7-MED8 co-transformed with the pGADT7 vector were included as controls. (C) Split-luc assays showing that MED8 can interact with FAMA in *N*. *benthamiana* leaves. Three biological replicates were performed, and similar results were obtained. (D) Co-IP assays were used to verify the interaction of MED8 with FAMA in *N*. *benthamiana* leaves. Protein extracts from *N*. *benthamiana* leaves infiltration with both *35Spro*:*FAMA-HA* and *35Spro*:*MED8-GFP* (*FAMA-HA MED8-GFP*) or *35Spro*:*GFP-myc* (*FAMA-HA GFP-myc*) was immunoprecipitated (IP) with the GFP antibody, and immunoprecipitated proteins were analyzed by immunoblotting using anti-HA and anti-GFP antibodies. The experiments were repeated three times, with similar results.

Previous research demonstrated that MED25 could interact with the transcription factor MYC2 to act as part of the general transcriptional machinery in regulating JA-triggered gene expression [16]. However, the results from the yeast two-hybrid assay indicated no interaction between MED8 and MYC2 (Fig 2A). This corroborates previous results whereby MED8 and MED25 influenced JA-mediated pathogen resistance by independent and additive mechanisms [11], leading us to speculate that the interaction of another potential partner with MED8 might be involved in pathogen resistance. To test this, we used the Y2H system to identify potential factors that could interact with MED8. The full-length MED8 was fused to the Gal4 DNA binding domain of the bait vector (BD-MED8). After screening, three independent clones encoding FAMA, which plays an indispensable role in plant stomatal development [30], were identified by prototrophy for His and Ade. The full-length coding sequence (CDS) of FAMA was introduced into the prey vector (AD-FAMA), and the bait and prey vectors were co-transformed into yeast for reconstructing the protein–protein interaction (Fig 2B). A further split-luciferase complementation (Split-LUC) assay was performed in *N*. *benthamiana* leaves to validate the Y2H assay results. As illustrated in Fig 2C, the co-expression of nLUC-tagged MED8 with cLUC-FAMA produced detectable luciferase activity, confirming the results of the Y2H assay. In contrast, the co-expression of nLUC/cLUC, nLUC/cLUC-FAMA, or MED8-nLUC/cLUC resulted only in background luciferase signals (Fig 2C). In addition to the Split-LUC assays, MED8-FAMA interaction was verified by Co-IP assays using *N*. *benthamiana* total protein (Fig 2D). Taken together, these results indicate that MED8 interacts with FAMA proteins in the plant cell, implying that MED8 might regulate pathogen resistance by interacting with FAMA.

### *fama* loss-of-function mutants attenuate the defense response against *B*. *cinerea*

**Fig 3:**
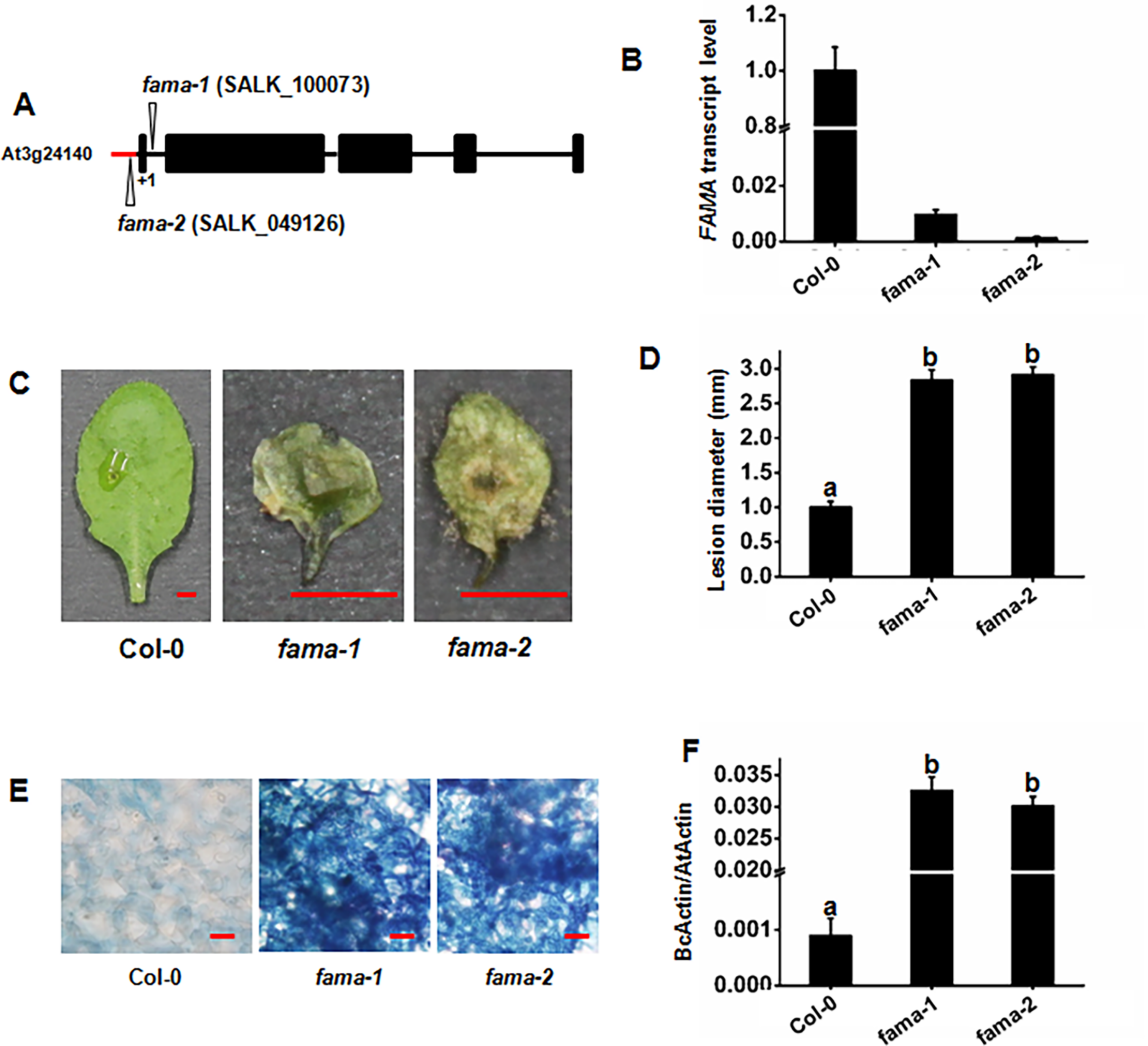
*fama* mutants display increased susceptibility to *B*. *cinerea* infection. (A) Gene organization of *FAMA*. T-DNA insertions are shown for *fama-1* (SALK_100073) and *fama-2* (SALK_049126). Closed boxes, exons; black solid lines, introns; red solid lines, 5’ UTR; The translational start sites (ATG) are shown as +1. (B) RT-qPCR of *FAMA* at 14 d after germination of *fama-1* and *fama-2*, and their respective wild-type lines (Col-0) using *Actin7* as a control. Error bars indicate 95% confidence intervals (n = 3). (C) (D) Disease symptoms and lesion sizes on the *B*. *cinerea*-infected WT, *fama-1*, and *fama-2* leaves. (E) Trypan blue staining of *B*. *cinerea* fungal hyphae growing on leaves at 2 d. Bars = 100 μm. (F) Fungal growth on the *B*. *cinerea*-infected WT, *fama-1*, and *fama-2* leaves. The disease assay was performed as indicated (see Results and Methods) on the leaves of soil-grown plants. Photos (C) were taken at 2 d. Bars = 2.5 mm. Fungal growth *in planta* was assumed by analyzing the transcript levels of the *BcActinA* gene by RT-qPCR using *Actin7* as an internal control 2 d after inoculation (F). Average values and SEM from relative values obtained in three biological replicates were plotted on the graph (D) (F). A minimum of 10 leaves for each genotype was used for each biological replicate, and the disease assay was repeated three times, with similar results. The mean values followed by different letters represent significant differences (*P*< 0.01, Student’s *t*-test).

FAMA was previously shown to influence stomatal cell fate [30]. Our results demonstrated that it could interact with MED8, suggesting that FAMA is probably involved in plant immunity towards *B*. *cinerea*. In order to investigate the function of FAMA in pathogen resistance, the phenotypes of two *fama* loss-of-function mutants, *fama-1* (SALK_100073) and *fama-2* (SALK_049126), were evaluated. The mutation site of *fama-1* is located in the first intron of the *FAMA* gene, while that of *fama-2* is in the promoter region of *FAMA* (Fig 3A). Both of the mutants failed to accumulate *FAMA* transcripts (Fig 3B). The disease phenotypes of the mutants after inoculation with *B*. *cinerea* were evaluated. The *fama* mutants were shown to display severe defects in growth, and healthy leaves of the *fama* mutants were chosen to compare the inoculation phenotypes (S1 Fig). In the detached leaf disease assays, *fama-1* and *fama-2* displayed rapid spreading of the pathogen. Two days after inoculation with *B*. *cinerea*, *fama-1* and *fama-2* showed severe and typical disease phenotypes for *B*. *cinerea*, while the WT control plants only possessed small spots on their leaves (Fig 3C and 3D). Further whole plant disease assays were carried out to confirm the disease phenotype observed in the *fama-1* and *fama-2* plants. In the whole plant disease assays, the two mutants plants along with the WT plants were inoculated by spraying with *B*. *cinerea* spore suspension. The disease phenotypes were revealed by trypan blue staining of fungal hyphae and *in planta* fungal growth was analyzed by comparing the transcription of the *B*. *cinerea* actin gene *BcActin* as an indicator of fungal growth *in planta*. The results indicated that *fama-1* and *fama-2* displayed much denser fungal hyphal growth than that of WT. Furthermore, the RT-qPCR determination of fungal growth also showed that *fama-1* and *fama-2* were susceptible to *B*. *cinerea* (Fig 3E and 3F). To summarize, our disease experiments demonstrate that the *fama* loss-of-function mutants exhibit an enhanced susceptibility towards *B*. *cinerea* infection.

### *FAMA*-overexpressed plants exhibit enhanced resistance to *B*. *cinerea* infection

**Fig 4:**
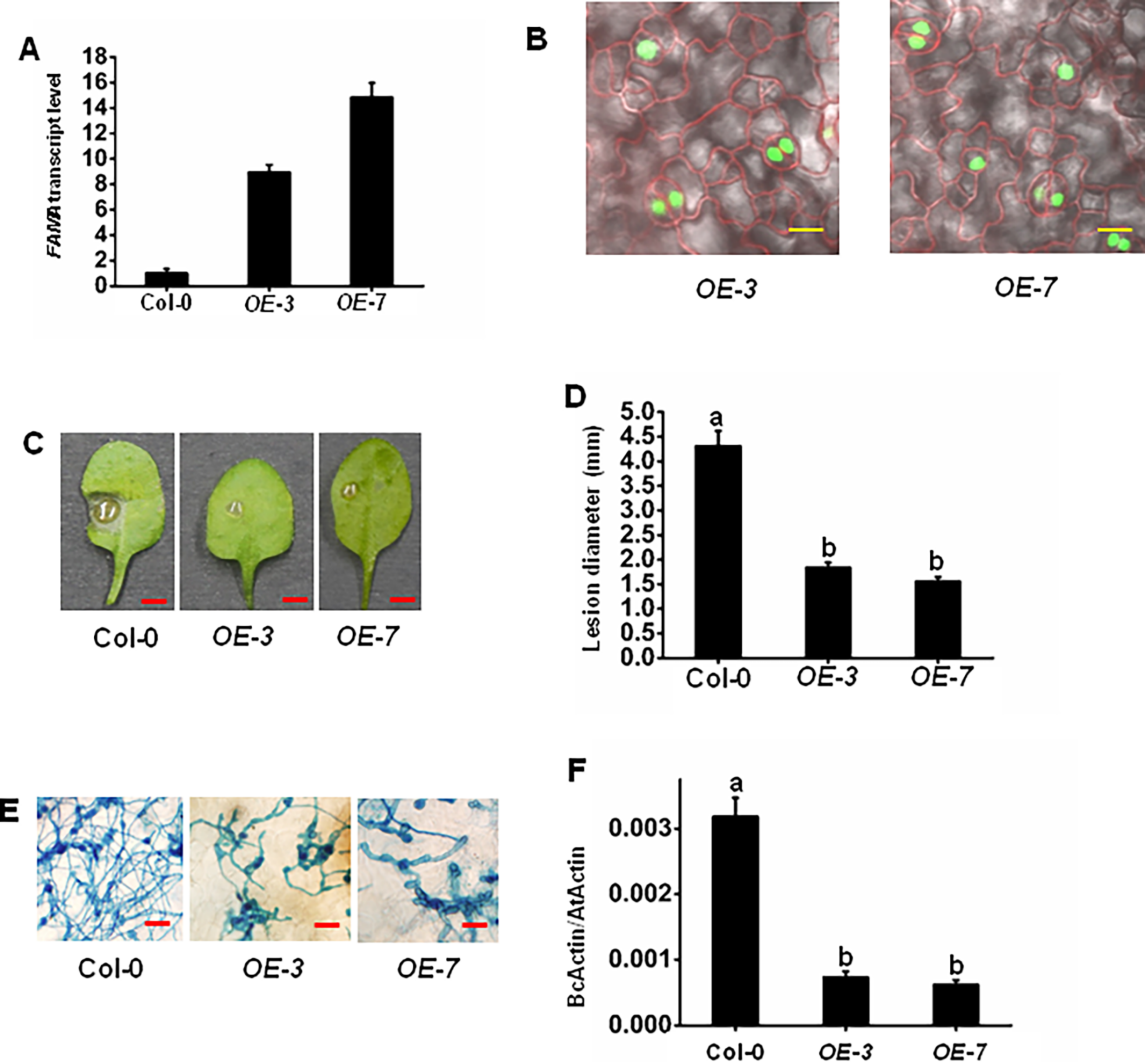
*FAMA* overexpression plants enhance *B*. *cinerea* resistance. (A) RT-qPCR of *FAMA* at 6 d after germination of *FAMA* overexpression lines (*OE-3* and *OE-7*) and WT using *Actin7* as a control. Error bars indicate 95% confidence intervals (n = 3). (B) Localization of FAMA in *OE-3* and *OE-7* leaves. Confocal imaging of transgenic *A*. *thaliana* plants expressing *FAMA*. Bars = 20 μm. (C) (D) Disease symptoms and lesion sizes on the *B*. *cinerea*-infected WT, *OE-3*, and *OE-7* leaves. (E) Trypan blue staining of *B*. *cinerea* fungal hyphae growing on leaves at 3 d. Bars = 100 μm. (F) Fungal growth on the *B*. *cinerea*-infected WT, *OE-3*, and *OE-7* leaves. The disease assay was performed as indicated (see Results and Methods) on the leaves of soil-grown plants. Photos (C) were taken at 3 d. Bars = 3 mm. Fungal growth *in planta* was assumed by analyzing the transcript levels of the *BcActinA* gene by RT-qPCR using *Actin7* as an internal control 3 d after inoculation (F). Average values and SEM from relative values obtained in three biological replicates were plotted on the graph (D) (F). A minimum of 10 leaves for each genotype was used for each biological replicate, and the disease assay was repeated three times, with similar results. The mean values followed by different letters represent significant differences (*P*< 0.01, Student’s *t*-test).

To further explore the role of *FAMA* in pathogen resistance, *FAMA*-overexpressed plants were generated and their immunity phenotypes toward *B*. *cinerea* infection were evaluated. Two *FAMA* overexpression lines *OE-3* (*ProFAMA*: *FAMA-GFP* 3#) and *OE-7* (*ProFAMA*: *FAMA-GFP* 7#) generated significantly higher *FAMA* transcript levels than WT as revealed by RT-qPCR (Fig 4A). *FAMA* was found to express in the stomatal guard cells as determined by fluorescence microscopy (Fig 4B), which is in accordance with previous results [30]. Detached leaf disease assays of *B*. *cinerea* in overexpressed lines and WT suggested that FAMA is a positive regulator of *B*. *cinerea* resistance, as evidenced by the significant alleviation of disease symptoms in the *OE-3* and *OE-7* lines and obvious disease symptoms in WT, including larger and expanding disease lesions beyond the inoculation site (Fig 4C and 4D). Fungal hyphae stained with trypan blue and RT-qPCR determination of fungal growth also indicated that the *OE-3* and *OE-7* lines were resistant to *B*. *cinerea* infection (Fig 4E and 4F). In combination, these data further demonstrate that FAMA positively influences plant immunity towards *B*. *cinerea*.

### FAMA affects pathogen-induced plant defensin gene expression

**Fig 5:**
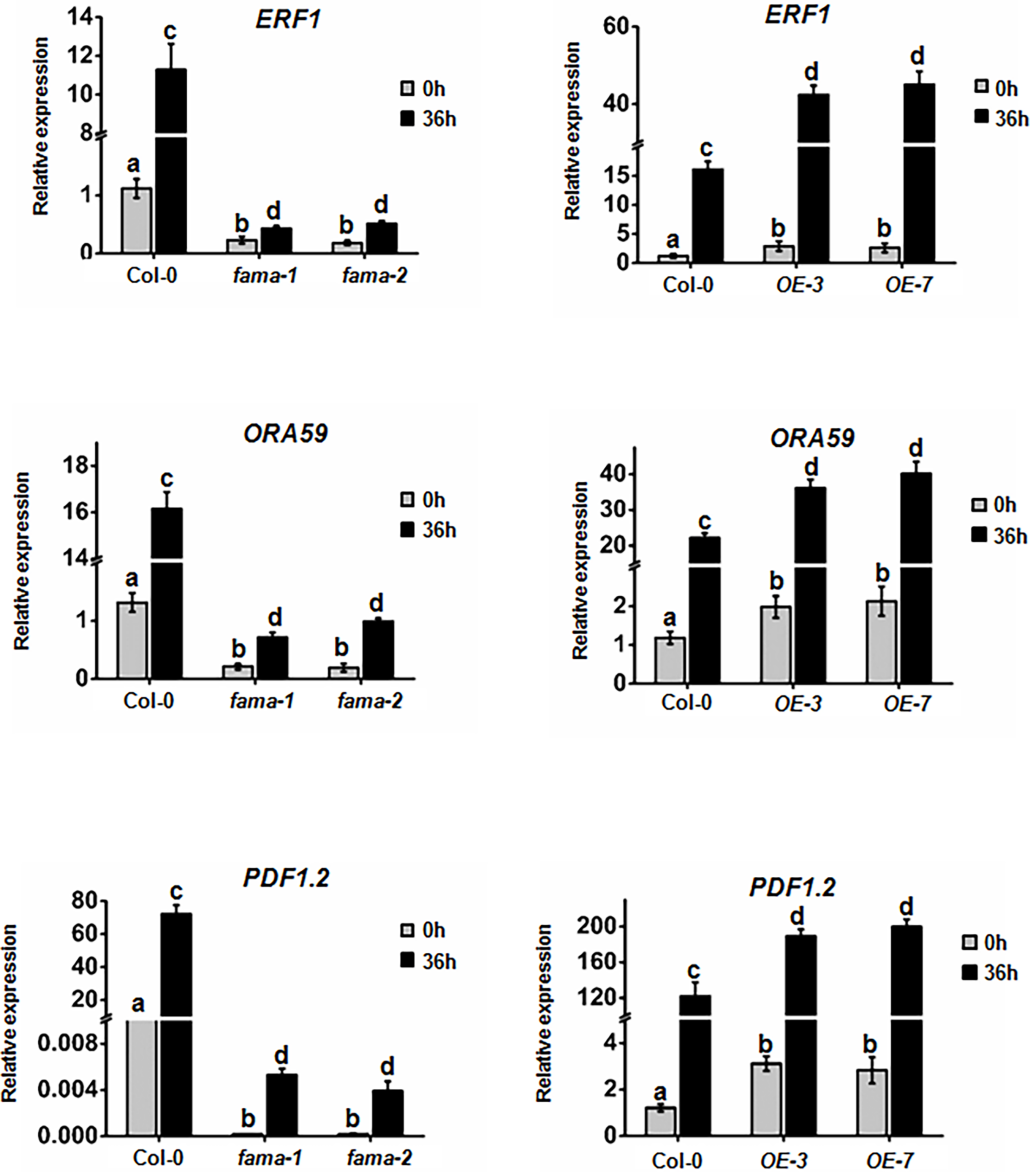
FAMA affects the function of MED8 in regulating the transcriptional expression of pathogen-responsive genes. Expression of *ERF1*, *ORA59*, and *PDF1*.*2* was examined by RT-qPCR in Col-0, *fama-1*, *fama-2*, *OE-3*, and *OE-7* plants following inoculation of *B*. *cinerea*. Average values and SEM from relative values obtained in three biological replicates were plotted on the graph. A minimum of 10 leaves for each genotype was used for each biological replicate, and the disease assay was repeated three times, with similar results. The mean values followed by different letters represent significant differences (*P*< 0.01, Student’s *t*-test).

The expression levels of three JA-induced plant defensin genes (*ERF1*, *ORA59*, and *PDF1*.*2*) were examined in *FAMA* mutants and overexpression lines. The results demonstrated that the mRNA levels were significantly reduced in the *fama-1* and *fama-2* plants compared to WT plants following *B*. *cinerea* inoculation. On the contrary, the expression of *ERF1*, *ORA59*, and *PDF1*.*2* were increased in *OE-3* and *OE-7* overexpression plants at 36 h after inoculation with *B*. *cinerea*. Additionally, the basal levels of *ERF1*, *ORA59*, and *PDF1*.*2* were also relatively lower in *fama-1* and *fama-2* plants in comparison with WT plants, but higher in *OE-3* and *OE-7* overexpression plants in comparison with WT plants (Fig 5). Furthermore, the expression of one salicylic acid (SA)-induced plant pathogenesis-related (*PR1*) gene was also examined in *FAMA* mutants and overexpression lines. In contrast to the three JA-induced plant defensin genes, the expression of *PR1* was reduced in *OE-3* and *OE-7*, but increased in the *fama-1* and *fama-2* plants before and after *B*. *cinerea* inoculation (S2 Fig). In combination, our gene expression results thus indicate that FAMA influences pathogen resistance at both basal and induced levels.

### FAMA and MED8 work together in pathogen resistance

**Fig 6:**
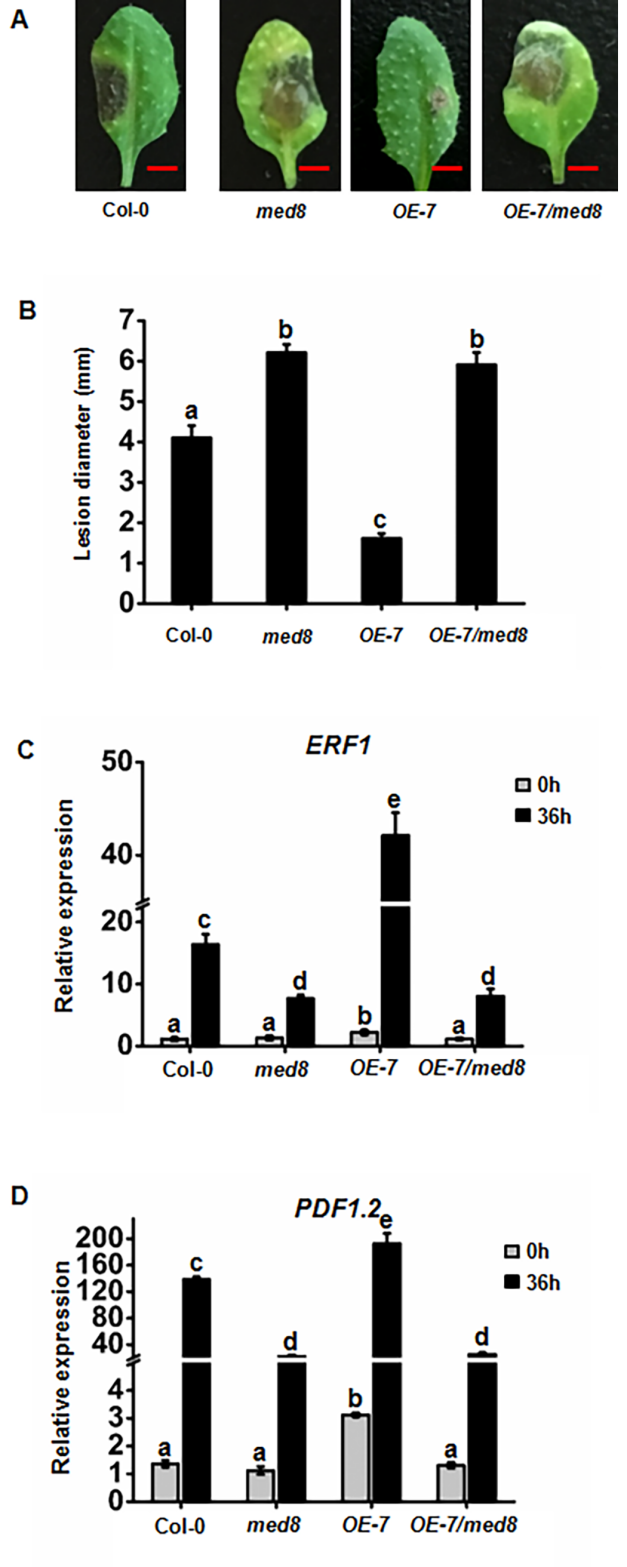
FAMA-activated defense responses are MED8 dependent. (A) (B) Disease symptoms and lesion sizes of the *B*. *cinerea*-infected WT, *med8*, *OE-7*, and *OE-7/med8* leaves at 3 d. The disease assay was performed by drop inoculation of *B*. *cinerea* on the leaves of soil-grown plants. The infected leaves were photographed and bar = 4 mm (A). Average values and SEM from relative values obtained from three biological replicates were plotted on the graph (B). A minimum of 10 leaves for each genotype was used for each biological replicate, and the disease assay was repeated in triplicate, with similar results. The mean values followed by different letters represent significant differences (*P*< 0.01, Student’s *t*-test). (C) (D) RT-qPCR analysis of *ERF1* and *PDF1*.*2* RNA levels in the WT, *med8*, *OE-7*, and *OE-7/med8* leaves of soil-grown plants at 36 hpi after inoculation with *B*. *cinerea*. Expression of *ERF1* and *PDF1*.*2* was normalized against the constitutively expressed *Actin7*. Average values and SEM from relative values obtained in three replicates were plotted on the graph. A minimum of 10 leaves for each genotype was used for each biological replicate, and the disease assay was repeated three times, with similar results. The mean values followed by different letters represent significant differences (*P*< 0.01, Student’s *t*-test).

The above results demonstrate that MED8 and FAMA are required for plant immunity against *B*. *cinerea* infection (Fig 1A; Fig 3C; Fig 4C). We also found that FAMA could directly interact with MED8 (Fig 2B), implying that FAMA and MED8 might operate in the same pathogen resistance pathway. To test this hypothesis, the *med8fama-2* double mutant was constructed. The pathogen inoculation results showed that MED8 and FAMA acted in the same plant immunity pathway during *B*. *cinerea* infection (S3 Fig). We also crossed the previously characterized *ProFAMA*: *FAMA-GFP* transgene into the *med8* mutant background and investigated the associated effects in pathogen resistance. Confirming our previous observation, *med8* was susceptive to *B*. *cinerea* infection, while *OE-7* showed enhanced resistance to *B*. *cinerea* (Fig 6A and 6B). However, the effects of *FAMA* overexpression on pathogen resistance were completely blocked by the *med8* mutant (Fig 6A and 6B), indicating that *FAMA*-activated pathogen resistance requires MED8. Expression of the plant defensin genes *ERF1* and *PDF1*.*2* further supported that *FAMA*-mediated resistance against *B*. *cinerea* depends on MED8 (Fig 6C and 6D).

### MED8 and FAMA bind the G-box region of the promoter of *ORA59*

**Fig 7:**
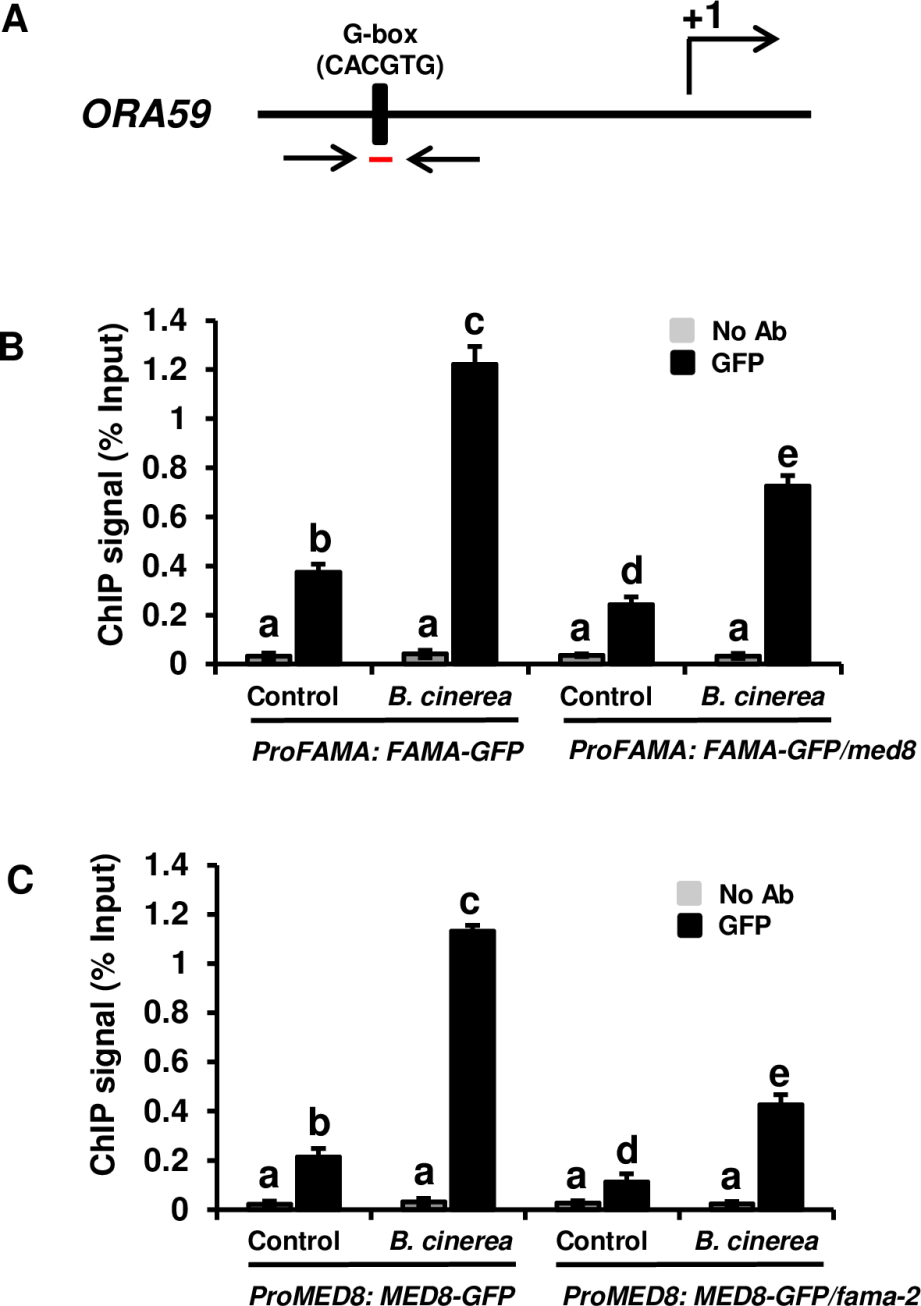
FAMA and MED8 can occupy the G-box region in the promoter of *ORA59*. (A) Schematic diagram of the promoter regions of *ORA59*. The black line represents the promoter region of the gene. The black box on the line indicates the putative G-box cis-elements (CACGTG) of the *ORA59* promoter. The region between the two coupled-arrowheads (red line) indicates the DNA fragments used for the ChIP-PCR. The translational start sites (ATG) are shown as +1. (B) FAMA could occupy the G-box region in the promoter of *ORA59*, and MED8 affects the recruitment of FAMA to the promoter of *ORA59*. The *ProFAMA*: *FAMA-GFP* and *ProFAMA*: *FAMA-GFP /med8* transgenic seedlings were used in ChIP using an anti-GFP antibody (Millipore). *ProFAMA*: *FAMA-GFP* and *ProFAMA*: *FAMA-GFP /med8* seedlings were inoculated with *B*. *cinerea* for varying lengths of time (0 and 36 h) before cross-linking. The “No Ab” immunoprecipitates served as negative controls. The ChIP signal was quantified as the percentage of total input DNA by RT-PCR. Average values and SEM from relative values obtained in four biological replicates were plotted on the graph. The ChIP assay was repeated at least four times, with similar results. The mean values followed by different letters represent significant differences (*P*< 0.01, Student’s *t*-test). (C) MED8 could occupy the G-box region in the promoter of *ORA59*, and FAMA affects the recruitment of MED8 to the promoter of *ORA59*. The *ProMED8*: *MED8-GFP* and *ProMED8*: *MED8-GFP /fama-2* transgenic seedlings were inoculated with *B*. *cinerea* for varying lengths of time (0 and 36 h) before cross-linking. The “No Ab” immunoprecipitates served as negative controls. The ChIP signal was quantified as the percentage of total input DNA by RT-PCR. Average values and SEM from relative values obtained from four biological replicates were plotted on the graph. The ChIP assay was repeated at least four times, with similar results. The mean values followed by different letters represent significant differences (*P*< 0.01, Student’s *t*-test).

*ERF1* and *PFD1*.*2* constitute downstream genes in plant immunity to *B*. *cinerea*. The RT-qPCR results showed that the expression levels of *ERF1* and *PFD1*.*2* changed significantly in *fama* mutants and *FAMA-*overexpressed plants. The ability of MED8 and FAMA to directly occupy the *ERF1* or *PFD1*.*2* promoter was also assessed. ChIP assays using *ProMED8*: *MED8-GFP* and *ProFAMA*: *FAMA-GFP* plants and anti-GFP antibodies could not detect any significant occupation of MED8 and FAMA in the promoters of *ERF1* and *PFD1*.*2*. We then evaluated whether *ORA59*, which occupies the promoter of *PFD1*.*2* [51], could be the direct target of MED8 and FAMA. The ChIP assays indicated that MED8 and FAMA had bound to the G-box region in the promoter of *ORA59*, which is also the occupation site of MYC2 at steady state [16]. We also found that *B*. *cinerea* inoculation resulted in a marked increase in the binding of MED8 and FAMA within 36 h (Fig 7B and 7C), while the non-G-box region in the promoter of *ORA59* exhibited little MED8 and FAMA enrichment neither at steady state nor after *B*. *cinerea* inoculation (S4 Fig). These data suggest that *ORA59* might constitute the direct target of MED8 and FAMA.

We also assessed the interaction between FAMA and MED8 during binding the promoter of the target gene *ORA59*. For this purpose, we crossed the previously characterized *ProFAMA*: *FAMA-GFP* transgene into the *med8* mutant background (*ProFAMA*: *FAMA-GFP/med8*). In *ProFAMA*: *FAMA-GFP/med8* plants, the recruitment of FAMA to the promoter of *ORA59* was severely reduced whether inoculated with *B*. *cinerea* or not (Fig 7B). This result indicated that MED8 could influence the recruitment of FAMA to the promoter of *ORA59*. We also assessed whether FAMA could influence the recruitment of MED8 to the promoter of *ORA59*. We crossed the *ProMED8*: *MED8-GFP* transgene into the *fama-2* mutant background (*ProMED8*: *MED8-GFP / fama-2*) and discovered that the recruitment of MED8 to the promoter of *ORA59* was also reduced in *ProMED8*: *MED8-GFP / fama-2* plants, demonstrating that the recruitment of MED8 to the promoter of *ORA59* is FAMA-dependent (Fig 7C).

### FAMA directly activates the expression of *ORA59*

**Fig 8:**
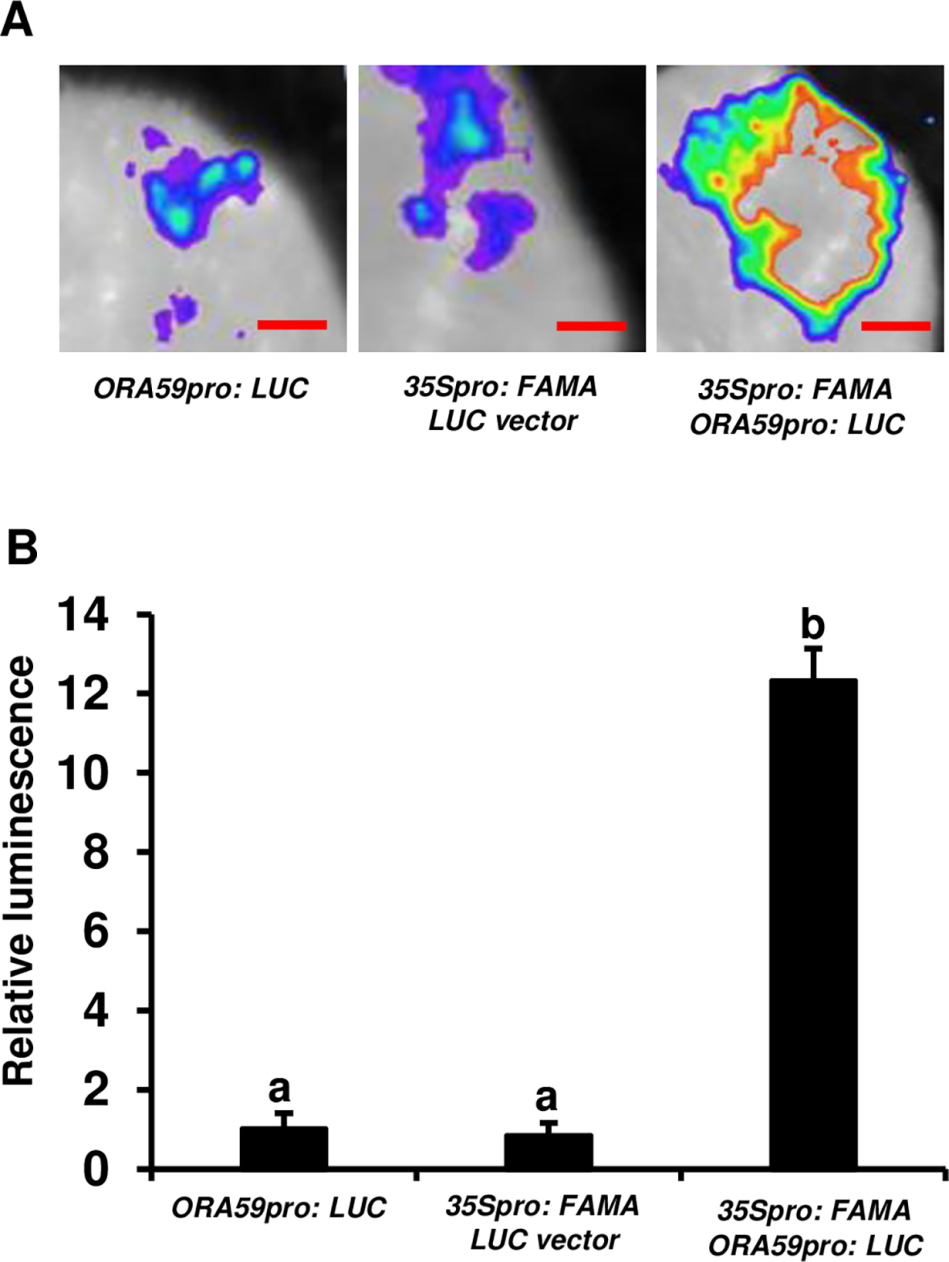
FAMA can activate the expression of *ORA59*. (A) Transient expression assays showing that the overexpression of *FAMA* could activate *ORA59* expression. Representative images of *N*. *benthamiana* leaves 72 h after infiltration are shown. The bottom panel indicates the infiltrated constructs. Bars = 2.5 mm. (B) Quantitative analysis of luminescence intensity in (A). Average values and SEM from relative values obtained in four biological replicates were plotted on the graph. The transient expression assay was repeated at least four times, with similar results. The mean values followed by different letters represent significant differences (*P*< 0.01, Student’s *t*-test).

ChIP-PCR assays using *ProFAMA*: *FAMA-GFP* plants indicated that FAMA had bound to the promoter of *ORA59*. To test whether FAMA could directly activate the expression of *ORA59*, we used a transient assay to compare the activatory effect of FAMA on the expression of *ORA59*_*pro*_: *LUC* reporters containing the *ORA59* promoter fused with the *LUC* gene. The co-expression of *ORA59*_*pro*_: *LUC* with *35S*_*pro*_: *FAMA* led to an obvious increase in luminescence intensity, indicating that *35S*_*pro*_: *FAMA* activated the expression of *ORA59*_*pro*_: *LUC*. In contrast, expression of the empty LUC vector with *35S*_*pro*_: *FAMA* or *ORA59*_*pro*_: *LUC* alone resulted in little luciferase signal (Fig 8A and 8B). These results imply that FAMA is required for the expression of *ORA59*.

## Discussion

The transcriptional regulation of gene expression plays a central role in plant immunity [52, 53]. Upon pathogenic infection, plant cells trigger genome-wide transcriptional reprogramming [54, 55]. Increasing efforts are being made to elucidate the functions of the Mediator complex, which interacts with RNA polymerase during gene transcription in plant immunity. For example, the Arabidopsis WRKY33 transcription factor-activated transcription of *PDF 1*.*2* and *ORA59* depends on mediator subunit MED16 for resistance to the pathogenic fungus *Sclerotinia sclerotiorum* [25]. Similarly, in this study, we provide evidence that MED8 regulates *Botrytis* resistance through interaction with FAMA transcription factor.

MED8 in combination with MED25 was initially reported to be involved in the defense response of plants to a variety of biotic stresses by independent and additive mechanisms [11]. The knockdown of *NtMed8*, which is homologous to the MED8 subunit of the *Arabidopsis* Mediator complex, caused abnormal development of the vegetative and floral organs in tobacco [56]. A later study found that MED8 could regulate organ size in *Arabidopsis* [57]. Recently, MED8 was shown to be necessary for the transcriptional regulation of genes associated with cell elongation and cell wall composition in response to cell wall defects and in sugar-responsive gene expression [58]. These results suggest that the *Arabidopsis* MED8 subunit has multiple roles in the development and stress response of plants. However, in contrast to the *Arabidopsis* Mediator subunit MED25, the molecular mechanisms underlying the diverse functions of MED8 are largely unclear.

**Fig 9:**
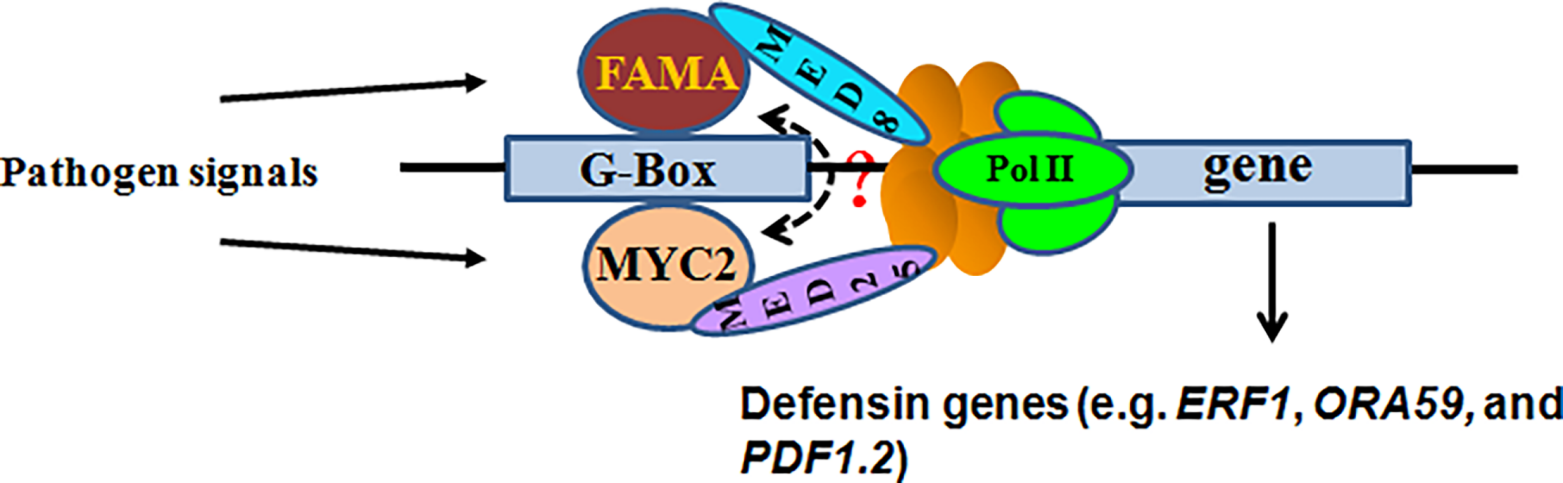
A model of FAMA-MED8 mediated signaling pathway in pathogen resistance. Once pathogen signals are perceived by plant cells, FAMA will recruit MED8 to the G-box region in the promoter of *FAMA* targets. MED8 then recruits pol II to the promoter of *FAMA* target and activates downstream defensin genes. This signaling pathway is independent of the MYC2-MED25 signaling pathway in pathogen resistance.

Our investigation of the function of MED8 in *Botrytis* resistance focused on its interplay with FAMA; a basic helix-loop-helix (bHLH) transcription factor that acts a key component in stomatal development [30]. Our speculation that MED8 regulates plant immunity via its interaction with FAMA is based on the following findings. Firstly, MED8 interacted directly with FAMA (Fig 2B–2D), suggesting that MED8 and FAMA might function together in the same pathway. Secondly, a novel function for FAMA in plant immunity to *B*. *cinerea* was revealed in this study (Figs 3C and 4C), which indicated that MED8 could be recruited by FAMA during *B*. *cinerea* infection. Thirdly, genetic analyses revealed that MED8 affected the functioning of FAMA in the regulation of pathogen resistance and the expression of pathogen-responsive genes (Fig 6A–6F). Most importantly, we discovered that MED8 could be recruited to the promoter region of the FAMA target gene (Fig 7A–7C). Based on the above results, our study presents a new FAMA-MED8 mediated *Botrytis* resistance pathway. As shown in Fig 9, FAMA will bind the G-box region in the promoter of *ORA59* when the pathogen signals are perceived by plant cells. FAMA will then recruit MED8 to the promoter of its target and activate the expression of downstream defensin genes, such as *PDF1*.*2*. The above-described mode of action of the *Arabidopsis* MED8 together with FAMA in regulating *Botrytis* resistance is similar to that of the *Arabidopsis* Mediator subunit MED25 in regulating MYC2-mediated transcription. It has been shown that in the presence of JA, MED25 is recruited to the promoter regions of MYC2 targets and, through direct interaction with MYC2, positively influences MYC2 transcriptional regulation [16]. The Mediator complex is considered to connect gene-specific transcription factors with RNA polymerase machinery to regulate gene expression [59]. Chen et al., showed that MED25 mediated the recruitment of the Pol II subunit to the promoter of MYC2 targets [16]. Wang et al., also demonstrated that MED16 played a key role in JA/ ethylene (ET)-induced recruitment of RNAPII to *PDF1*.*2* and *ORA59* [25]. As a subunit of the Mediator complex, we speculate that MED8 is likely to recruit Pol II to the promoter of the FAMA target and regulate its expression. However, this speculation requires further experimental verification. Collectively, these results illustrate that MED8 might act as a coactivator of FAMA in regulating *Botrytis* resistance.

The necrotrophic pathogen *B*. *cinerea* can infect the host via cuticle penetration. In addition to the enzymolysis holes caused by necrotrophic pathogens, pathogens may enter the plant via the stomata, which serve as passive ports of entry during infection [60]. Therefore, it follows that defective stomata could significantly prevent pathogen infection, while increased stomatal numbers could promote pathogen infection. However, the *FAMA* mutation associated with defective stomata exhibits reduced resistance to *B*. *cinerea* infection, whereas the overexpression of *FAMA* results in a stoma-in-stoma (SIS) phenotype, characterized by the asymmetrical division of guard cells, which confers increased resistance to *B*. *cinerea* infection. This implied that the positive role of FAMA in *B*. *cinerea* resistance might be stomata-independent. Accordingly, we discovered that the expression of defense genes was significantly affected in *FAMA* mutated or overexpression plants. Additionally, FAMA was found to occupy the promoter of *ORA59* along with MED8 upon *B*. *cinerea* infection, suggesting that FAMA regulates *B*. *cinerea* resistance through affecting defense gene expression other than the reason of developmental alteration. This differs slightly from that observed in *NtMEK2*. Under dexamethasone induction, *GVG-Nt-MEK2*^*DD*^ plants exhibited no stomatal differentiation on the cotyledon epidermis, but were resistant to *B*. *cinerea* infection [61, 62]. These results suggest a complex relationship between stomatal development and *B*. *cinerea* resistance.

Our study revealed for the first time that FAMA functions in plant immunity by mediating the expression of defense genes. FAMA occupies the promoter of *ORA59* along with MED8 upon *B*. *cinerea* infection. However, it was not able to directly occupy the promoter of *PDF1*.*2*. Similar results were observed with the MYC2 transcription factor in the JA pathway. MYC2 mediates JA-mediated *B*. *cinerea* resistance by regulating the expression of the MYC2-target gene, *ORA59* [51]. However, MYC2 has not been found to directly occupy the promoter of *PDF1*.*2* in any of the relevant literature, which suggests that transcription factors regulate gene expression at different levels. MYC2, as a core transcription factor in the JA pathway, mediates two branches of the JA pathway by targeting different downstream transcription factors. MYC2 can occupy the promoters of *NAC019* and *NAC055* to positively regulate JA-mediated root growth and insect resistance, and also occupy the promoter of *ORA59* to negatively regulate JA-mediated pathogen resistance [51, 63]. Similarly, FAMA can occupy the promoters of the transcription factor *bHLH090* in myrosin cell development and *ORA59* in pathogen resistance [40]. This indicates that the upstream core transcription factors could regulate different specific downstream transcription factors to mediate specific pathways. Although both MYC2 and FAMA can bind the promoter of *ORA59* to regulate *B*. *cinerea* resistance, they display opposite phenotypes in *B*. *cinerea* resistance [64]. Song et al., showed that MYC2 interacted with EIN3 to attenuate the transcription of *ORA59* and repress ET-enhanced *B*. *cinerea* resistance [65]. Conversely, our results showed that FAMA could induce the expression of *ORA59* and positively regulate the transcription of *PDF1*.*2*. An assessment of the genetic relationship between FAMA and MYC2 in *B*. *cinerea* resistance is warranted.

Our data demonstrate that MED8 regulates plant immunity towards *B*. *cinerea* by interacting with the transcription factor FAMA. Both MED8 and FAMA could occupy the G-box region in the promoter of *ORA59* following *B*. *cinerea* inoculation (Fig 7A–7C). However, several questions regarding the mechanisms of action of MED8 and FAMA in pathogen resistance remain unanswered. Further identification of the genes regulated by both MED8 and FAMA should elucidate the molecular mechanisms and the signaling pathways involved in the regulation of pathogen resistance by MED8 and FAMA. Particularly, the identification of genes regulated by both MED8 and FAMA in other signaling pathways should enhance our understanding of the functions of MED8 and FAMA. Elucidating the genetic relationships of FAMA and MYC2 is important for clarifying the molecular mechanisms of FAMA in plant immunity toward *B*. *cinerea*.

## Supporting information

S1 Fig The morphological phenotypes of indicated mutants and the transgenic lines. Photographs of Col-0, *fama-1*, *fama-2*, *OE-3*, *OE-7*, *med8*, and *med25* plants were taken three to four weeks after being grown on soil without *B*. *cinerea* inoculation. Bars = 1 cm.
(TIF)

S2 Fig FAMA affects the transcriptional expression of SA-induced *PR1* gene. Expression of *PR1* was examined by RT-qPCR in Col-0, *fama-1*, *fama-2*, *OE-3*, and *OE-7* plants following inoculation of *B*. *cinerea*. Average values and SEM from relative values obtained in four biological replicates were plotted on the graph. A minimum of 10 leaves for each genotype was used for each biological replicate, and the disease assay was repeated at least four times, with similar results. The mean values followed by different letters represent significant differences (*P*< 0.01, Student’s *t*-test).
(TIF)

S3 Fig *med8fama-2* displays increased susceptibility to *B*. *cinerea* infection. (A) (B) Disease symptoms and lesion sizes on the *B*. *cinerea*-infected WT, *med8*, *fama-2*, and *med8fama-2* leaves at 2 days.
The disease assay was performed by drop inoculation of *B*. *cinerea* on the leaves of soil-grown plants. The infected leaves were photographed and bar = 2.5 mm (A). Average values and SEM from relative values obtained from three biological replicates were plotted on the graph (B). A minimum of 10 leaves for each genotype was used for each biological replicate, and the disease assay was repeated three times, with similar results. The mean values followed by different letters represent significant differences (*P*< 0.01, Student’s *t*-test).
(TIF)

S4 Fig FAMA and MED8 specifically bind the G-box region in the promoter of *ORA59*. (A) Schematic diagram of the promoter regions of *ORA59*. The black line represents the promoter region of the gene. The black box on the line and red line with the letter “a” indicates the putative G-box cis-elements (CACGTG) of the *ORA59* promoter; the red line with letter “b” indicates the non-G-box region of the *ORA59* promoter. The regions of “a” and “b” indicate the DNA fragments used for ChIP-PCR. The translational start sites (ATG) are shown as +1.
(B) FAMA could occupy the G-box region in the promoter of *ORA59*, but not the non-G-box region in the promoter of *ORA59*. The *ProFAMA*: *FAMA-GFP* transgenic seedlings were used in ChIP using an anti-GFP antibody (Millipore). *ProFAMA*: *FAMA-GFP* seedlings were inoculated with *B*. *cinerea* for varying lengths of time (0 and 36 h) before cross-linking. The “No Ab” (no antibody) immunoprecipitates served as negative controls. The ChIP signal was quantified as the percentage of total input DNA by real-time PCR. Three biological replicates were performed and identical results were obtained. Standard deviations were calculated from 3 technical replicates.
(C) MED8 could occupy the G-box region in the promoter of *ORA59*, but not the non-G-box region in the promoter of *ORA59*. The *ProMED8*: *MED8-GFP* transgenic seedlings were used in ChIP using an anti-GFP antibody (Millipore). *ProMED8*: *MED8-GFP* seedlings were inoculated with *B*. *cinerea* for varying lengths of time (0 and 36 h) before cross-linking. The “No Ab” (no antibody) immunoprecipitates served as negative controls. The ChIP signal was quantified as the percentage of total input DNA by real-time PCR. Three biological replicates were performed and identical results were obtained. Standard deviations were calculated from 3 technical replicates.
(TIF)

S1 Table Primers used in this study. (PDF)
