# Supplementary figures and images for: The Arabidopsis thaliana Mediator subunit MED8 regulates plant immunity to Botrytis Cinerea through interacting with the basic helix-loop-helix (bHLH) transcription factor FAMA
Source: PLoS One. 2018 Mar 7;13(3):e0193458. doi: 10.1371/journal.pone.0193458 (PMC5841781; doi:10.1371/journal.pone.0193458)

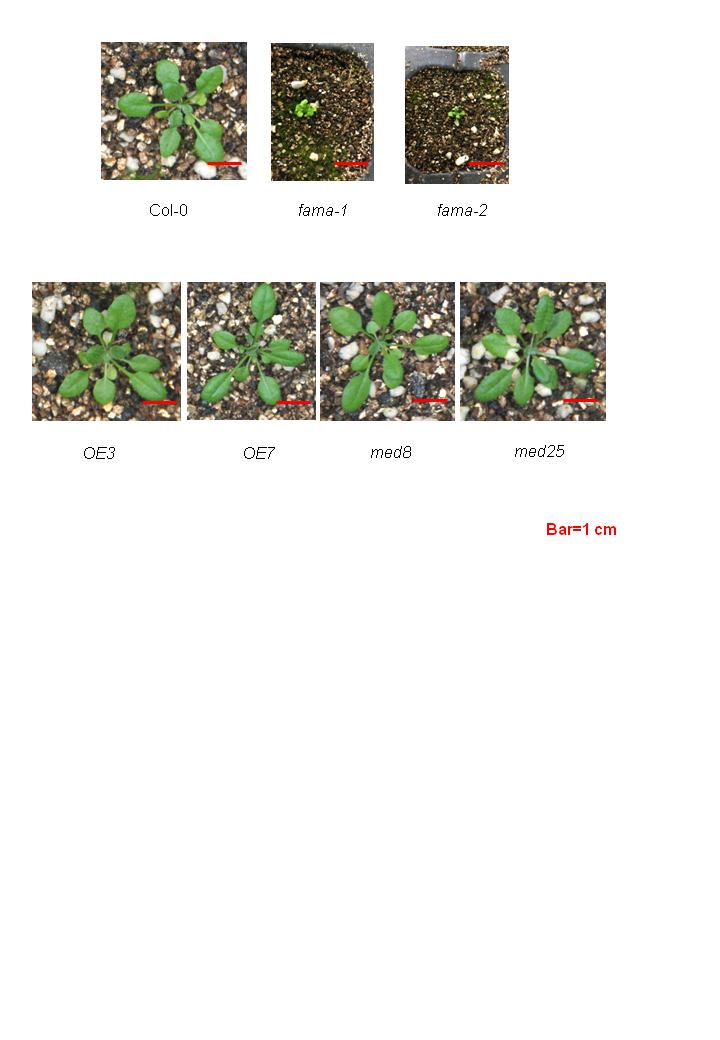

Supplement: S1 Fig — Photographs of Col-0, fama-1, fama-2, OE-3, OE-7, med8, and med25 plants were taken three to four weeks after being grown on soil without B. cinerea inoculation. Bars = 1 cm. (TIF) [file pone.0193458.s001.tif]

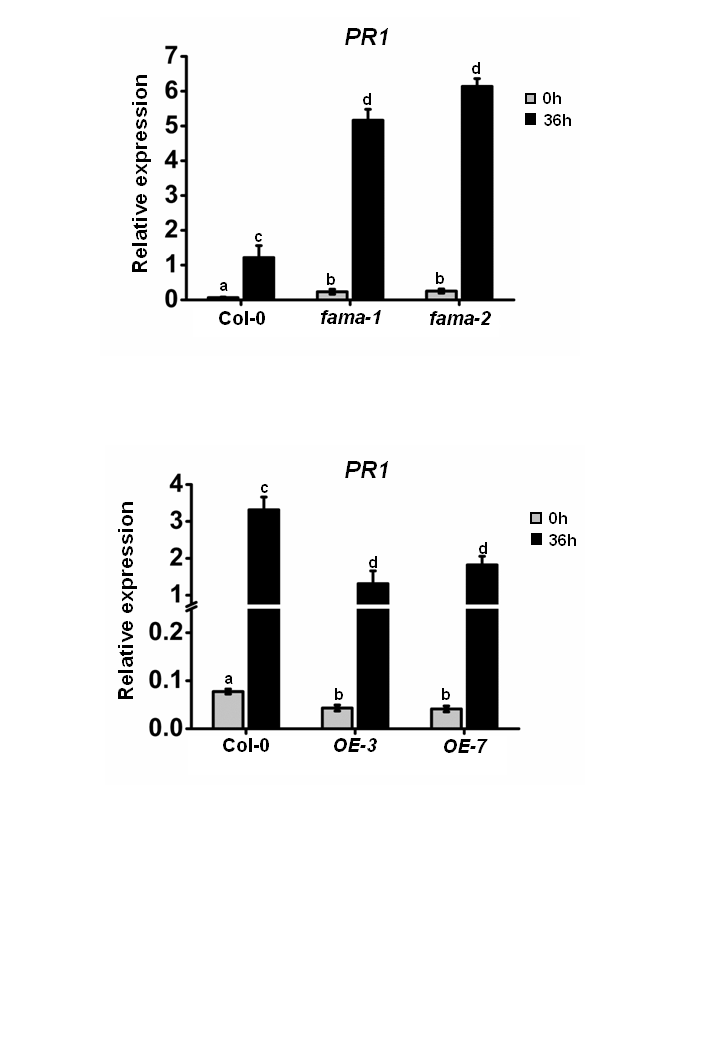

Supplement: S2 Fig — Expression of PR1 was examined by RT-qPCR in Col-0, fama-1, fama-2, OE-3, and OE-7 plants following inoculation of B. cinerea. Average values and SEM from relative values obtained in four biological replicates were plotted on the graph. A minimum of 10 leaves for each genotype was used for each biological replicate, and the disease assay was repeated at least four times, with similar results. The mean values followed by different letters represent significant differences (P< 0.01, Student’s t-test). (TIF) [file pone.0193458.s002.tif]

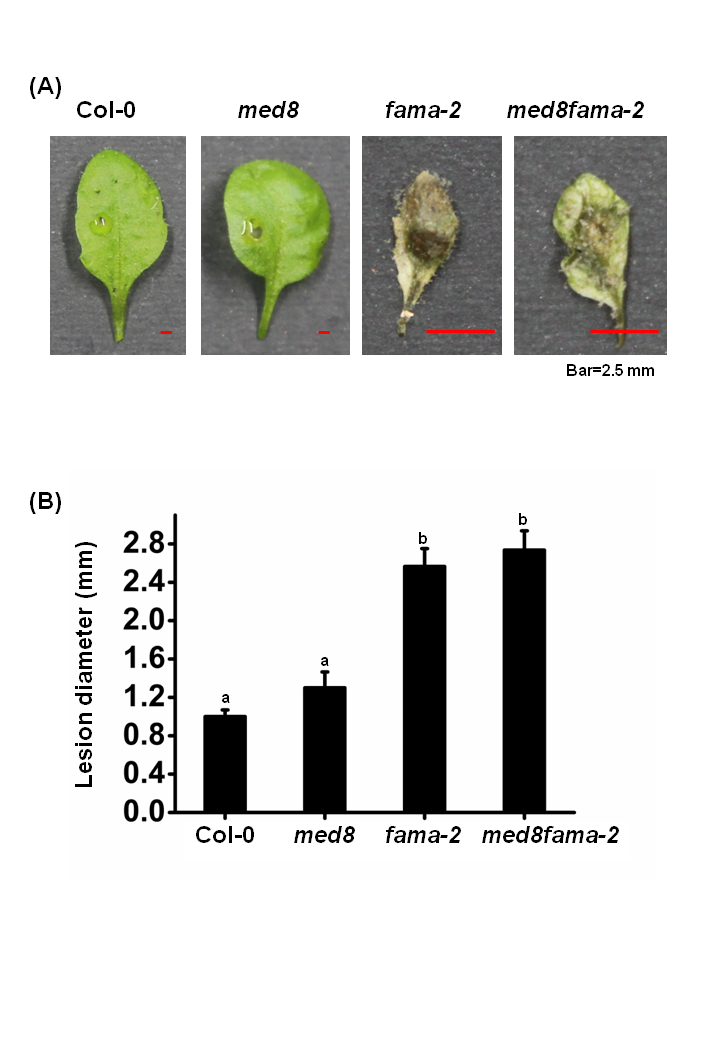

Supplement: S3 Fig — (A) (B) Disease symptoms and lesion sizes on the B. cinerea-infected WT, med8, fama-2, and med8fama-2 leaves at 2 days. The disease assay was performed by drop inoculation of B. cinerea on the leaves of soil-grown plants. The infected leaves were photographed and bar = 2.5 mm (A). Average values and SEM from relative values obtained from three biological replicates were plotted on the graph (B). A minimum of 10 leaves for each genotype was used for each biological replicate, and the disease assay was repeated three times, with similar results. The mean values followed by different letters represent significant differences (P< 0.01, Student’s t-test). (TIF) [file pone.0193458.s003.tif]

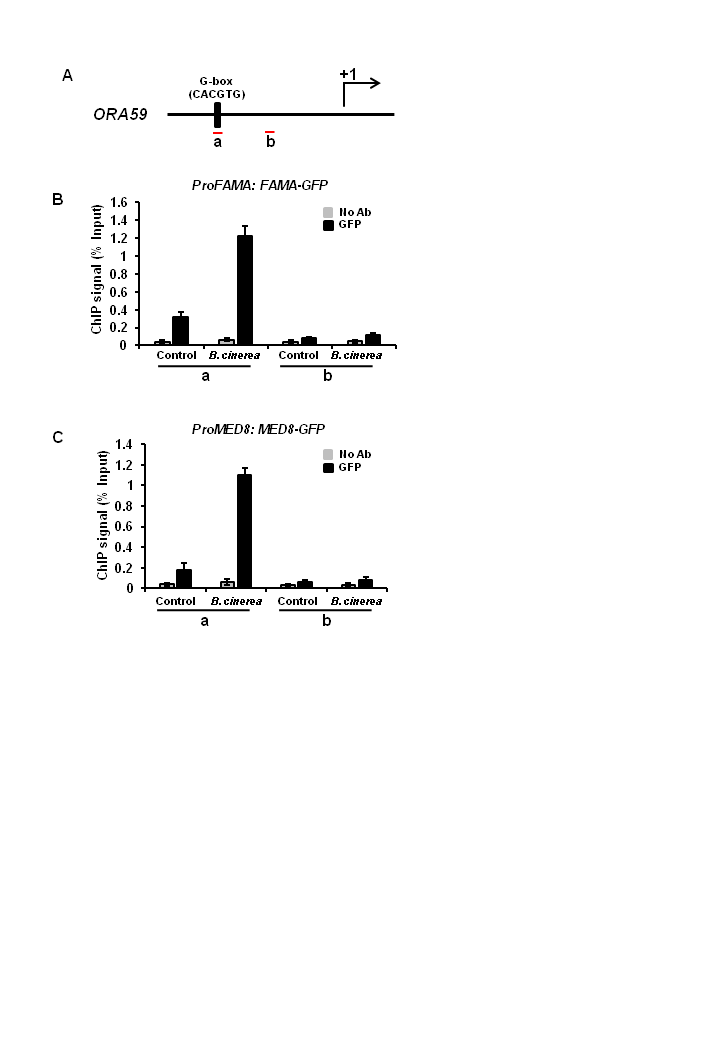

Supplement: S4 Fig — (A) Schematic diagram of the promoter regions of ORA59. The black line represents the promoter region of the gene. The black box on the line and red line with the letter “a” indicates the putative G-box cis-elements (CACGTG) of the ORA59 promoter; the red line with letter “b” indicates the non-G-box region of the ORA59 promoter. The regions of “a” and “b” indicate the DNA fragments used for ChIP-PCR. The translational start sites (ATG) are shown as +1. (B) FAMA could occupy the G-box region in the promoter of ORA59, but not the non-G-box region in the promoter of ORA59. The ProFAMA: FAMA-GFP transgenic seedlings were used in ChIP using an anti-GFP antibody (Millipore). ProFAMA: FAMA-GFP seedlings were inoculated with B. cinerea for varying lengths of time (0 and 36 h) before cross-linking. The “No Ab” (no antibody) immunoprecipitates served as negative controls. The ChIP signal was quantified as the percentage of total input DNA by real-time PCR. Three biological replicates were performed and identical results were obtained. Standard deviations were calculated from 3 technical replicates. (C) MED8 could occupy the G-box region in the promoter of ORA59, but not the non-G-box region in the promoter of ORA59. The ProMED8: MED8-GFP transgenic seedlings were used in ChIP using an anti-GFP antibody (Millipore). ProMED8: MED8-GFP seedlings were inoculated with B. cinerea for varying lengths of time (0 and 36 h) before cross-linking. The “No Ab” (no antibody) immunoprecipitates served as negative controls. The ChIP signal was quantified as the percentage of total input DNA by real-time PCR. Three biological replicates were performed and identical results were obtained. Standard deviations were calculated from 3 technical replicates. (TIF) [file pone.0193458.s004.tif]
