# Supplementary material for: The Arabidopsis thaliana Mediator subunit MED8 regulates plant immunity to Botrytis Cinerea through interacting with the basic helix-loop-helix (bHLH) transcription factor FAMA
Source: PLoS One. 2018 Mar 7;13(3):e0193458. doi: 10.1371/journal.pone.0193458 (PMC5841781; doi:10.1371/journal.pone.0193458)
Supplement: S1 Table — (PDF) [file pone.0193458.s005.pdf]

Supplemental Table 1. The primers used in this study.

**Primers for Y2H**	
**MED8-BD**	**F:**GGAATTCCATATGATGGAGACACAGCCGCAGCAACC
	**R:**CCGGAATTCTTATTGAGGATTTTGGTGCCTTTG
**MED25-BD**	**F:**CGGAATTCATGTCGTCGGAGGTGAAACAG
	**R:**TTGGATCCTTATCCCATGAAGCCAGCTCC
**FAMA-AD**	**F:**GGAATTCCATATGATGGATAAAGATTACTCGGCAC
	**R:**CGCGGATCCTCAAGTAAACACAATATTTCCC
**MYC2-AD**	**F:**GGGAATTCCATATGACTGATTACCGGCTACAACCAACGATGA
	**R:**CCATCGATCACCGATTTTTGAAATCAAACTTGC
**Primers for qRT-PCR**	
**ERF1-RT**	**F:** GGAAATTCGCGGCGGAGATAAGA
	**R:** CAACGCCACAACCGGAGAACAAC
**PDF1.2-RT**	**F:**CGCACCGGCAATGGTGGAAG
	**R:**CACACGATTTAGCACCAAAG
**ORA59-RT**	**F:**AAGGGATAAGAGTGTGGCTTGG
	**R:**CCGGAGAGATTCTTCAACGACA
**PR1-RT**	**F:** TCGTCTTTGTAGCTCTTGTAGGTG
	**R:** TAGATTCTCGTAATCTCAGCTCT
**BcActin-RT**	**F:** CGTCACTACCTTCAACTCCATC
	**R:** CGGAGATACCTGGGTACATAGT
**ACT7-RT**	**F:**CCATTCAGGCCGTTCTTTC

	**R:**CGTTCTGCGGTAGTGGTGA
**ORA59-a-CHIP**	**F:**CATCTATTTTGTCGGGAATAC
	**R:**GGTGCTACAATAATAATATTC
**ORA59-b-CHIP**	**F:** CGTCTCTCTTGCGCTACAGGTT
	**R:** GGATGAGCCTATTAGTGGTGGACC
**Primers for transgene vectors construction**	
**FAMA**	**F:**ATGGATAAAGATTACTCGGCAC
	**R:**TCAAGTAAACACAATATTTCCC
**MED8**	**F:**ATGGAGACACAGCCGCAGCAACC
	**R:**TTATTGAGGATTTTGGTGCCTTTG
**FAMA-CDS-1300**	**F:**CGCGGATCCATGGATAAAGATTACTCGGCAC
	**R**:CGGGGTACCAGTAAACACAATATTTCCCAGGT
**FAMA-Promotor-1300**	**F:**TGCACTGCAGTATATCAATCAGTCTCTTCGCT
	**R:**GCGTCGACTGCTATTCGTGGTAGTTGATTATAA
**MED8-CDS-1300**	**F:**CGCGGATCCATGGAGACACAGCCGCAGCAACC
	**R:**CGGGGTACCTTGAGGATTTTGGTGCCTTTG
**MED8-Promotor-1300**	**F:**TGCACTGCAGAGAAACTCTCTCTTCCTTCCC
	**R:**GCGTCGACTGATAACACAGAGCTAATACAC
**Primers for LUC experiments**	
**FAMA-cLUC**	**F:**CGGGGTACCATGGATAAAGATTACTCGGCAC
	**R:**AACTGCAGTCAAGTAAACACAATATTTCCC
**MED8-nLUC**	**F:**CGGGGTACCATGGAGACACAGCCGCAGCAACC

	**R:**ACGCGTCGACTTATTGAGGATTTTGGTGCCTTTG
**ORA59$_{Pro}$-LUC**	**F:**CCCAAGCTTAATTTGGTTAGGCTAGGGACG
	**R:**TAACTGCAGTTTCGATCTTTTTTTTTTCTTC
